# Draft genome sequences of two *Vibrio jasicida* strains isolated from the Atlantic brief squid *Lolliguncula brevis*

**DOI:** 10.1128/mra.00963-26

**Published:** 2026-10-06

**Authors:** Steph Smith, Alecia Septer

**Affiliations:** 1 Department of Earth, Marine, and Environmental Sciences, University of North Carolina at Chapel Hill 2331, Chapel Hill, North Carolina, USA; 2 Institute of Marine Sciences, University of North Carolina at Chapel Hill, Chapel Hill, North Carolina, USA; California State University San Marcos, San Marcos, California, USA

**Keywords:** *Vibrio*, symbiosis, whole genome sequencing

## Abstract

We report draft genome sequences of two *Vibrio jasicida* strains, LB10LO4 and LB14LO7, cultured from tissues of the Atlantic brief squid *Lolliguncula brevis* in Bogue Sound, North Carolina, USA.

## ANNOUNCEMENT

Members of the genus *Vibrio* (family Vibrionaceae) are widespread in the microbiota of marine animals, including cephalopods, where they act as beneficial symbionts, commensals, and opportunistic pathogens (1, 2). The Atlantic brief squid *Lolliguncula brevis* is a euryhaline cephalopod of shallow western-Atlantic estuaries, and diverse culturable *Vibrio* spp. have previously been isolated from this host (3). These include published genome sequences of *Vibrio campbellii* (strain LB10LO1) and *Vibrio fischeri* (strain LB10LO6) (4, 5). As part of an ongoing survey of *L. brevis*-associated vibrios, we sequenced two *Vibrio jasicida* strains, LB10LO4 and LB14LO7, cultured from mantle-cavity tissues of different animals.

Squid were collected by trawl from Bogue Sound, North Carolina, USA (34.64734°N, 76.65320°W) (12 June 2017). Mantle-cavity tissue was aseptically dissected, rinsed three times in sterile artificial seawater, and spread onto LBS agar; plates were incubated at 24°C for 24 h. Strain LB10LO4 was cultured from animal LB10 and strain LB14LO7 from animal LB14. Single colonies were purified by three successive streaks on LBS agar and preserved as glycerol stocks at −80°C.

Genomic DNA was extracted with the Zymo Fungal/Bacterial DNA Miniprep kit following the manufacturer’s protocol and submitted to Plasmidsaurus (Eugene, OR, USA) for long-read whole-genome sequencing on the Oxford Nanopore Technologies GridION platform. Libraries were prepared with the rapid barcoding kit SQK-RBK114.96 without shearing or size selection and sequenced on a FLO-MIN114 (R10.4.1) flow cell. Reads were basecalled with the super-accuracy model dna_r10.4.1_e8.2_400bps_sup@v4.2.0, which removes sequencing adapters; no separate adapter-trimming step was required. Sequencing produced 824,000 reads (3.38 Gb; median quality Q49.1; ~582 × depth) for LB10LO4 and 588,000 reads (2.76 Gb; median quality Q49.4; ~490 × depth) for LB14LO7. Reads were quality-filtered and downsampled to ~100× with Filtlong v0.2.1 (https://github.com/rrwick/filtlong), assembled *de novo* with Flye v2.9.1 (6), and polished with Medaka v1.7.2 (https://github.com/nanoporetech/medaka). Default parameters were used.

**Fig 1:**
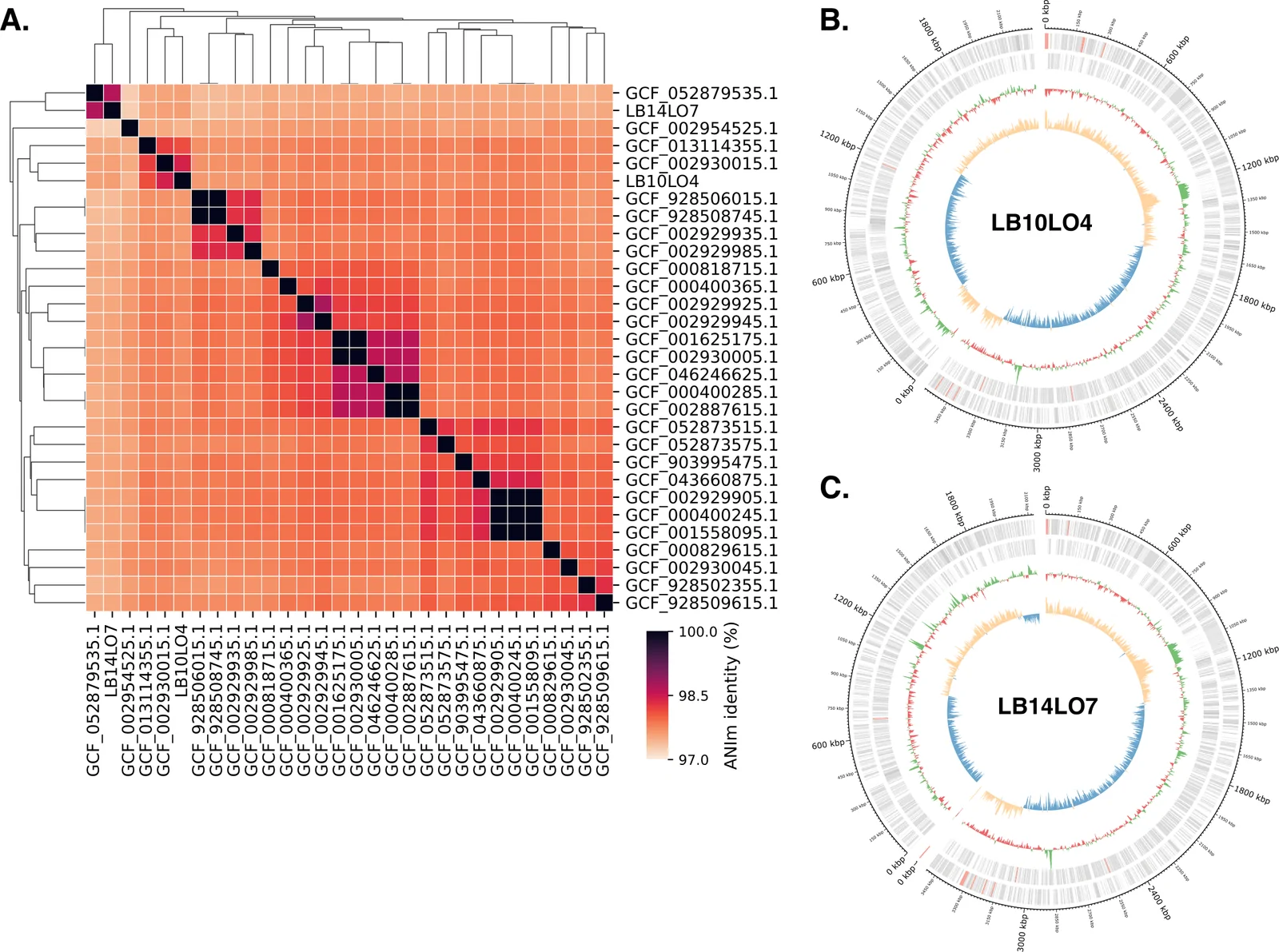
Genomic placement of *Vibrio jasicida* strains LB10LO4 and LB14LO7 from *Lolliguncula brevis*. (**A**) Heatmap of pairwise average nucleotide identity (ANIm) among 30 *V. jasicida* genomes, ordered by average-linkage. LB10LO4 and LB14LO7 fall within the species cluster (all pairs ≥ 97% ANI). Circular genome maps for LB10LO4 (**B**) and LB14LO7 (**C**). CDS on both strands, G + C content, and GC skew per replicon.

The LB10LO4 assembly comprised two circular contigs (5,814,810 bp; 45.2% G + C; largest 3,573,707 bp; ~101 × depth), and the LB14LO7 assembly comprised three circular contigs (5,630,229 bp; 45.0% G + C; largest 3,497,991 bp; ~100 × depth), the smallest a 5,537-bp putative plasmid (Fig. 1). Circularity was determined by Flye, which trims the terminal overlap of circular contigs; assemblies were not rotated to a fixed start position. The two large replicons of each strain are consistent with the characteristic two-chromosome architecture of *Vibrio*. CheckM v1.2.2 (7) estimated 100% completeness for both strains with 0.26% (LB10LO4) and 0.06% (LB14LO7) contamination. Genomes were annotated with the NCBI Prokaryotic Genome Annotation Pipeline (PGAP) v6.11 (8), yielding 5,243 genes (5,038 CDSs) for LB10LO4 and 5,069 genes (4,849 CDSs) for LB14LO7 (Table 1).

**TABLE 1:** Genome and sequencing statistics

Characteristics	LB10LO4	LB14LO7
Isolation		
Isolate species	*Vibrio jasicida*	*Vibrio jasicida*
Host species	*Lolliguncula brevis*	*Lolliguncula brevis*
Sequencing		
Sequencing platform	Oxford Nanopore GridION	Oxford Nanopore GridION
Total reads (raw)	824,000	588,000
Total bases sequenced (bp)	3,382,313,766	2,756,458,367
Median read length (bp)	2,771	3,160
Read N50 (bp)	12,514	13,458
Raw sequencing coverage (×)	582	490
Assembly depth (×)	101	100
Assembly		
Number of contigs	2	3
Total assembly length (bp)	5,814,810	5,630,229
Assembly N50 (bp)	3,573,707	3,497,991
G + C content (%)	45.2	45.0
Completeness (CheckM, %)	100	100
Contamination (CheckM, %)	0.26	0.06
Annotation		
Total genes	5,243	5,069
Protein-coding genes (CDS)	5,038	4,849
Pseudogenes	32	43
tRNAs	132	133
rRNAs	37	40
ncRNAs	4	4
GenBank accessions		
BioProject accession	PRJNA1497301	PRJNA1497301
BioSample accession	SAMN61797081	SAMN61797100
SRA accession	SRR39714211	SRR39714210
GenBank assembly accession	GCA_059772105.1	GCA_059772125.1

Species identity was confirmed as *V. jasicida* by Mash v2.3 (9) and Sourmash v4.6.1 (10) and by whole-genome average nucleotide identity (ANIm; pyANI v0.3.0 [11]). The closest Mash reference for both strains was *V. jasicida* 090810c (GCF_000400285.1). Both strains fell within a 30-genome *V. jasicida* cluster sharing ≥97% ANI and distinct from sister species *V. owensii* (Fig. 1). These genomes document the culturing of *V. jasicida*, alongside other vibrio species, from multiple *L. brevis* individuals and tissues.

## Data Availability

The whole-genome assemblies of *Vibrio jasicida* LB10LO4 and LB14LO7 have been deposited in GenBank under accession numbers GCA_059772105.1 and GCA_059772125.1, respectively, within BioProject PRJNA1497301. The strains correspond to BioSamples SAMN61797081 (LB10LO4) and SAMN61797100 (LB14LO7), and the raw Oxford Nanopore reads are available in the Sequence Read Archive under SRR39714211 and SRR39714210.
